# Epidemiology of Pregnancy Complications Through the Lens of Immunological Memory

**DOI:** 10.3389/fimmu.2021.693189

**Published:** 2021-06-25

**Authors:** Emily J. Gregory, James Liu, Hilary Miller-Handley, Jeremy M. Kinder, Sing Sing Way

**Affiliations:** ^1^ Department of Obstetrics and Gynecology, University of Cincinnati College of Medicine, Cincinnati, OH, United States; ^2^ Division of Infectious Diseases, Center for Inflammation and Tolerance, Cincinnati Children’s Hospital Medical Center, University of Cincinnati College of Medicine, Cincinnati, OH, United States

**Keywords:** parity, preeclampsia, prematurity, immunological memory, stillbirth

## Abstract

In the fifteen minutes it takes to read this short commentary, more than 400 babies will have been born too early, another 300 expecting mothers will develop preeclampsia, and 75 unborn third trimester fetuses will have died in utero (stillbirth). Given the lack of meaningful progress in understanding the physiological changes that occur to allow a healthy, full term pregnancy, it is perhaps not surprising that effective therapies against these great obstetrical syndromes that include prematurity, preeclampsia, and stillbirth remain elusive. Meanwhile, pregnancy complications remain the leading cause of infant and childhood mortality under age five. Does it have to be this way? What more can we collectively, as a biomedical community, or individually, as clinicians who care for women and newborn babies at high risk for pregnancy complications, do to protect individuals in these extremely vulnerable developmental windows? The problem of pregnancy complications and neonatal mortality is extraordinarily complex, with multiple unique, but complementary perspectives from scientific, epidemiological and public health viewpoints. Herein, we discuss the epidemiology of pregnancy complications, focusing on how the outcome of prior pregnancy impacts the risk of complication in the next pregnancy — and how the fundamental immunological principle of memory may promote this adaptive response.

## Introduction

The ability of immune cells to “remember” prior antigen encounters is a unifying principle in how we currently design vaccines. Prior infection with some microbial pathogens protects individuals against re-infection by the same pathogen. For example, a majority of individuals with resolved varicella infection are “immune” to re-infection by the varicella virus. Likewise, vaccination involves purposeful exposure to defined microbial antigens or attenuated pathogen variants. This exposure primes accumulation of pathogen-specific adaptive immune components that protects individuals against future infection.

A first step for evaluating whether this immunological lens of memory defined with vaccines and infection applied to pregnancy (exposure of mothers to genetically foreign paternal antigens expressed by the developing fetus), involves consideration of potential shifts in the incidence of complications in women during a first pregnancy compared with subsequent pregnancies, and the paternal-specificity of potential differences in susceptibility to pregnancy complications (1). Fetal tissues share equal genetic contribution from the mother and father, and are therefore semi-allogeneic to maternal immune components. An intricately orchestrated assortment of changes in the number, function and distribution of maternal immune cells occur in parallel with the anatomic and physiological shifts in women during pregnancy (2–4). These changes likely work together for averting premature rejection of fetal tissues, since perturbations in pregnancy induced maternal immune cell shifts are increasingly associated with a variety of pregnancy complications including preterm birth and spontaneous abortion (5–7). A classic example is the progressive expansion of maternal immune suppressive regulatory CD4+ T cells in women throughout pregnancy, and blunted expansion of these cells in women with preeclampsia (8–10).

## Parity and Immune Tolerance Memory in Preeclampsia and Other Pregnancy Complications

Preeclampsia occurs in 2-8% of pregnancies and is associated with systemic maternal inflammation and consistently elevated serum levels of classical proinflammatory cytokines including TNF-α, IFN-γ, IL-2, IL-6 and IL-8 (11, 12). While the underlying pathogenesis of preeclampsia remains undefined, fractured fetal tolerance is likely an important causative factor since the only effective treatment in affected women is delivery of the fetus and all products of conception (13). Historically, preeclampsia was described as a disorder of first pregnancies, reflecting the remarkably reduced incidence in multiparous compared with nulliparous women. Analysis of the Swedish birth register containing >700,000 births between 1984-2004 showed the 4.1% risk of preeclampsia in first pregnancy was reduced to 1.7% in subsequent pregnancies (14). Interestingly, these protective benefits of prior pregnancy also appear to be partner specific since preeclampsia risk consistently rebounds in multiparous women with a change in paternity (15–17). However, as any obstetrician can attest, risk of multiparous pre-eclampsia is also increased exponentially when it is present in the first pregnancy (11, 18). The risk of preeclampsia was increased to 14.7% in the second pregnancy in women who had preeclampsia in the first pregnancy, and 31.9% for women who had preeclampsia in the previous two pregnancies in the aforementioned Swedish birth register (14).

The clinical ramifications of fractured tolerance to genetically foreign paternal antigens expressed by the developing fetus likely have far-reaching implications beyond that of preeclampsia. Given the necessity for sustained tolerance to fetal-expressed paternal allo-antigens throughout pregnancy, differences in the tempo or timing of when tolerance is disrupted may result in a variety of unique clinical manifestations. Fractured fetal tolerance in the first trimester may result in spontaneous abortion, whereas perturbations in later pregnancy may instead result in stillbirth. More acute disruptions in fetal tolerance may cause preeclampsia/eclampsia, while more tempered perturbations may instead cause preterm birth or growth-restricted fetuses.

Importantly, these seemingly distinct pregnancy complications are also epidemiologically linked by parity. For example, a >15-fold increased frequency of premature birth was found among women with preeclampsia in a prior pregnancy in the Swedish birth register (14). Likewise, analysis of the Norwegian birth register between 1996 and 2013 containing >700,000 women from their first to second pregnancy showed the risk of preterm preeclampsia is 4-7 times higher in women with prior preterm birth without preeclampsia compared with women with prior term birth (19). Other studies report that the risk of stillbirth is significantly increased in women with a history of preeclampsia or premature birth in prior pregnancy (20, 21), whereas preterm birth risk is also increased in women with a history of stillbirth in a prior pregnancy (22, 23).

More recent analysis of the Norwegian birth register containing >300,000 women with two successive singleton pregnancies between 1999-2015 identified preeclampsia, placental abruption, stillbirth, neonatal death, and fetal growth restriction in prior pregnancy to each be independent risk factors for preterm birth in the next pregnancy (24). Preterm birth in prior pregnancy was also identified as a risk factor for preeclampsia, placental abruption, and growth restriction in the next pregnancy. The protective benefits of prior uncomplicated pregnancy against complications in next pregnancy were also shown with the 5.9% incidence of preterm birth in first pregnancy reduced to 3.1% in second pregnancy (24). Interestingly, preterm birth incidence in second pregnancies were further reduced to 2.9% in cases of shared paternity, but increased to 4.5% in cases with a new partner in second pregnancy. Thus, the importance of overlapping paternal-fetal allo-antigen in first and second pregnancies for stimulating either protective or harmful memory that impacts pregnancy outcomes is consistently highlighted in human epidemiological data.

A larger meta-analysis including >1.5 million births from data spanning 1967 to 2013 comparing the risk of stillbirth, preterm birth and fetal growth restriction found women who experienced any of these disorders in prior pregnancy were at significantly increased risk for each disorder in subsequent pregnancy (25). For example, the risk of stillbirth was increased 3-fold in women with prior preterm (<34 weeks gestation) birth. Reciprocally, the risk of preterm birth was increased 2.8-fold for women with a history of stillbirth. Thus, through the lens of immunology, pregnancy proves to be an efficient physiological model of tolerance to paternal allo-antigens expressed by the developing fetus. In turn, preeclampsia, prematurity, stillbirth, spontaneous abortion and fetal growth restriction are likely not discrete entities, but more likely complications occurring through a continuum affected by gestational age and complex trade-offs between immunological tolerance and rejection of the fetal allograft. By extension, many of these pregnancy complications likely share disruptions in fetal immune tolerance as an underlying causal factor, each creating long-lasting immunological memory consequences that impact the outcome of future pregnancy.

## Synergy Between Human Epidemiology and Preclinical Pregnancy Models

Limitations in analyzing the epidemiology of human pregnancy that preclude more decisive conclusions regarding how parity impacts pregnancy outcomes should also be highlighted. These include unreliable reporting of pregnancy, variability in number of partners between individuals and inter-pregnancy intervals, exposure to potential pathogenic and commensal microbes, plus the enormous genetic variability amongst individuals that is further magnified when considering the additional heterogeneity of partner (and fetal) specific antigens encountered in each pregnancy. The ensuing antigenic diversity in outbred human populations also preclude straightforward evaluation of maternal immune components with fetal-specificity, with the exception of Y-chromosome encoded antigens in male offspring pregnancies (26). Further complicating the analysis of human pregnancy outcome data with regards to parity and immunological memory are reductions in the risk of preeclampsia and other pregnancy complications with increasing duration of sexual cohabitation (27, 28), underscoring the tolerogenic properties of seminal fluid alone with pregnancy (29, 30). In this regard, preclinical pregnancy models using defined inbred animal strains for breeding offer an instructive opportunity for overcoming some of these limitations, and the potential to uncover mechanistic insights as to how mothers may immunologically tolerate and remember fetal antigens encountered in prior pregnancy.

**Figure 1:**
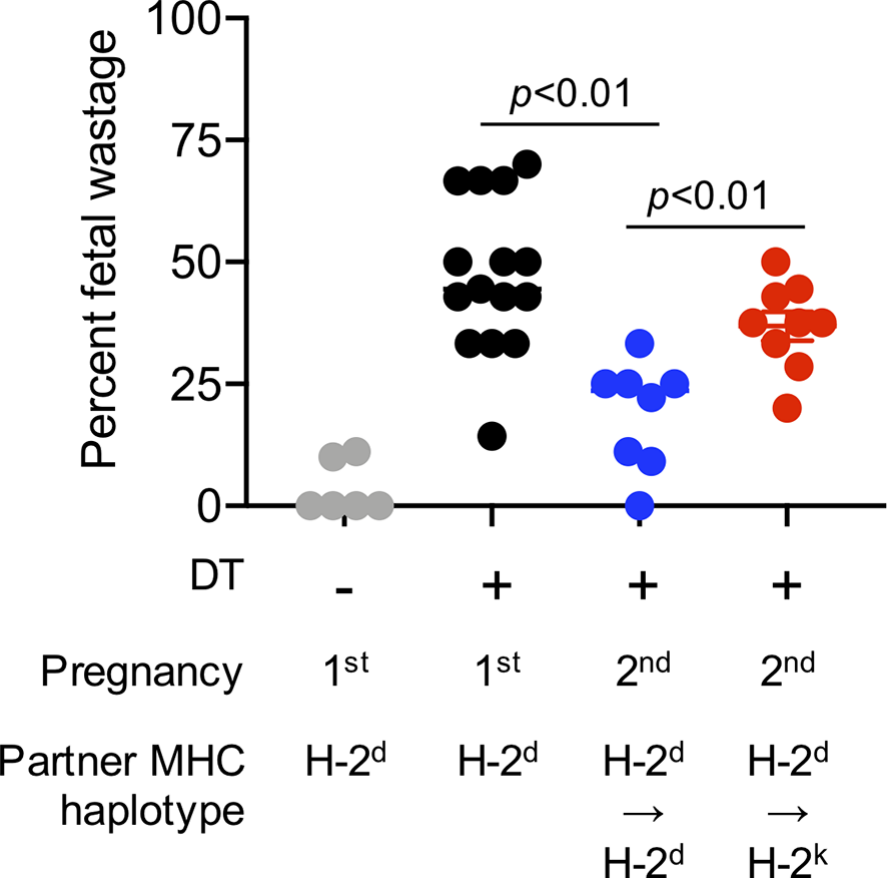
Primary pregnancy protects against maternal FOXP3+ regulatory CD4+ T cell depletion induced fetal wastage during second pregnancy in a partner specific fashion. Partial transient depletion of maternal FOXP3+ regulatory CD4+ T cells after diphtheria toxin treatment to FOXP3^DTR/WT^ heterozygous females has been described (31, 35). Percent fetal wastage for FOXP3^DTR/WT^ heterozygous females on the H-2^b^ C57BL/6 background administered purified diphtheria toxin (0.1 micrograms per dose intraperitoneal) for five consecutive days beginning midgestation (E10.5); and harvested at E15.5 during first pregnancy sired by H-2^d^ (Balb/c) male mice (black circles), compared with no diphtheria toxin treatment controls (gray), or during second pregnancy sired by H-2^d^ Balb/c male (blue) mice or second pregnancy sired H-2^k^ CBA male (red) mice.

Evaluating the importance of immune cells after experimental manipulation, and the impact on pregnancy outcomes is possible only in animal models. For example, fetal wastage in rodents efficiently occurs after experimental depletion of the aforementioned regulatory CD4+ T cells that normally expand in mothers during pregnancy (31, 32). Regulatory CD4+ T cell lineage specification is controlled by the X-linked transcription factor FOXP3 in both humans and mice (33). Regulatory CD4+ T cells are efficiently depleted following low-dose diphtheria toxin treatment to transgenic mice that co-express the high-affinity human diphtheria toxin receptor with FOXP3 (34). Using these transgenic mouse tools, we have previously shown that fetal wastage triggered by partial transient depletion of maternal FOXP3+ regulatory T cells is sharply more pronounced in primary compared with secondary pregnancy sired by genetically identical male partners (35). Importantly, these protective benefits are also partner specific since susceptibility to fetal wastage in second pregnancy rebounds when sired by MHC haplotype discordant “third party” male expressing distinct MHC haplotype antigens (**Figure 1**).

## Infection Induced Pregnancy Complications

Infection is another important consideration with regards to the pathogenesis of human pregnancy complications including preeclampsia, preterm birth and stillbirth (36–38). The microbes that cause infection can include commensal microbes, pathobiont microbes with the potential of invasive infection, microbes that cause latent infection with the potential for re-activation, and bona fide microbial pathogens that each represent sources of other genetically foreign antigens encountered by women during pregnancy. How does the maternal immune system distinguish between foreign microbial antigens expressed by commensal compared with pathogenic microbes, and further distinguish these microbial antigens from those expressed by the developing fetus?

Reductionist preclinical pregnancy models offer critical clues to these complex questions. For example, administration of live pathogen, purified microbial ligands and purified cytokines can each trigger preterm birth in rodents, and larger animals including non-human primates (39, 40). Recent studies highlight the unique transfer of antimicrobial peptides between decidual natural killer cells and trophoblast cells to sustain immunity at the maternal fetal interface (41). Increased susceptibility of women during pregnancy to specific prenatal pathogens, such as *Listeria monocytogenes*, is also recapitulated in animals where fetal wastage and congenital fetal invasion occur with experimental infection during pregnancy (31, 42, 43). Susceptibility to systemic *Listeria monocytogenes* infection is linked with expanded accumulation of maternal regulatory CD4+ T cells since depleting these cells at the expense of fracturing fetal tolerance overrides maternal infection susceptibility. In turn, infection and/or infection induced inflammation can also override the suppressive potency of regulatory T cells further causing disruptions in maternal-fetal tolerance (42, 44).

**Figure 2:**
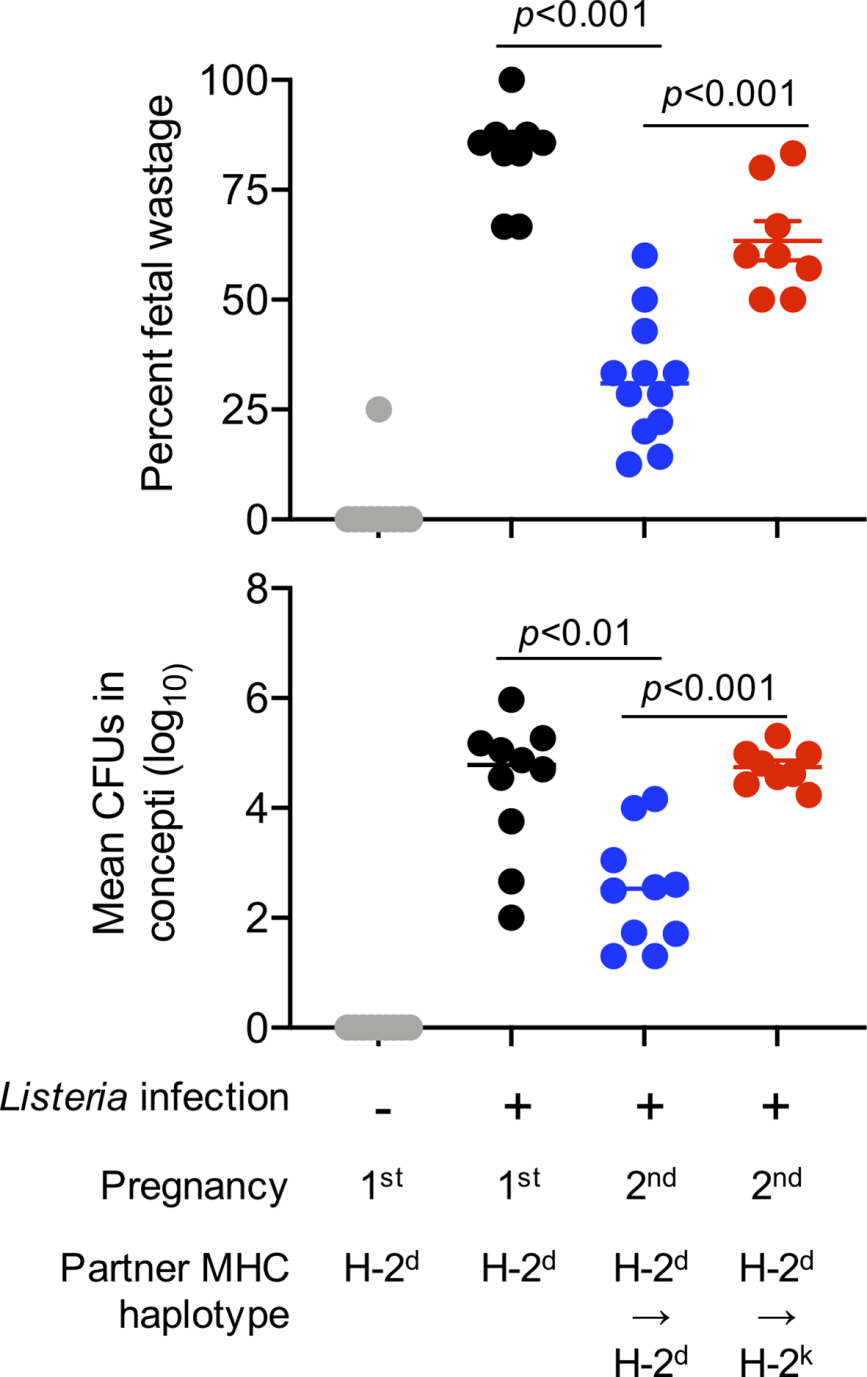
Primary pregnancy protects against prenatal *Listeria monocytogenes* infection induced fetal wastage during second pregnancy in a partner specific fashion. The immune-pathogenesis of *Listeria monocytogenes* prenatal infection induced fetal wastage has been described (42, 43). Percent fetal wastage (top) and mean recoverable bacterial CFUs for concept in each litter (bottom) for females on the H-2^b^ C57BL/6 background infected with *Listeria monocytogenes* strain 10403s (10^4^ CFUs administered intravenously) midgestation (E10.5); and harvested five days thereafter (E15.5) during first pregnancy sired by H-2^d^ (Balb/c) male mice (black circles), compared with no infection controls (gray), or during second pregnancy sired by H-2^d^ Balb/c male (blue) mice or second pregnancy sired H-2^k^ CBA male (red) mice.

Importantly, these infection-induced pregnancy complications can be used to further probe potential shifts in resiliency against complications between first and second pregnancies, and the potential partner specificity of these protective benefits. For example, *Listeria monocytogenes* infection induced fetal wastage and the degree of congenital fetal invasion are each significantly reduced in secondary compared with primary allogeneic pregnancies sired by genetically identical male mice, and changing paternity by using MHC haplotype discordant males to sire the second pregnancy overrides the protective benefits of prior pregnancy (**Figure 2**). Use of inbred strains with genetically identical homologous chromosomes in preclinical studies allows the MHC haplotype of offspring to be precisely controlled for in each mating, which is different from pregnancies in humans and other outbred species where with discordant homologous chromosomes (one maternally and one paternally-derived allele), the genetic makeup of fetal tissues, even in successive pregnancies with shared paternity, is likely to have unique antigenic features. This may explain why the protective benefits of prior pregnancy we find in mice are more consistent than benefits observed in humans. Together, these preclinical analyses that control for partner-specificity, inter-pregnancy interval and microbiota-pathogen specific features suggest that prior successful pregnancy protects against complications in next pregnancies and highlight the partner specificity of these protective impacts. Important areas for future investigation include evaluating whether similar parity and partner-specific protective benefits occur for pregnancy complications triggered by other prenatal antigenic exposures, antigenic overlap between fetuses (along with partner specificity) in successive pregnancies, and how cross-reactivity between microbe-specific T cells and trophoblast expressed allogeneic HLA impacts pregnancy outcomes (45).

## Maternal Innate and Adaptive Immune Cells With Fetal Specificity

An additional refinement to this approach involves siring pregnancies with transgenic male mice that ubiquitously express defined model antigens in non-transgenic female mice. This transforms model antigens into surrogate fetal antigens which allows for precise identification of maternal immune components with fetal-specificity (46). This approach shows massive expansion of maternal immune-suppressive FOXP3+ regulatory CD4+ T cells with fetal-specificity during primary pregnancy (35). Remarkably, these cells remain at expanded levels after parturition and re-accumulate with sharply more accelerated tempo after fetal antigen-restimulation during subsequent pregnancies. Likewise, primary pregnancy also primes systemic expansion of maternal CD8+ T cells with specificity to defined surrogate fetal antigens, and these cells persist at expanded levels after parturition (26, 47). Interestingly, and in sharp contrast to maternal memory FOXP3+ CD4+ T cells, maternal memory CD8+ T cells do not further expand with fetal-antigen restimulation during secondary pregnancy, but instead adopt a functionally exhausted phenotype synergistically mediated by high levels of cell-intrinsic PD-1 and LAG-3 expression (47). Thus, precise identification and tracking of maternal immune components with fetal specificity during pregnancy and after parturition using more reductionist preclinical models provide important immunological insights as to how mothers respond to genetically foreign paternal antigens in the context of mating and pregnancy, and how immunological memory that impacts the outcomes of next pregnancies may occur.

Function persistence of memory effector T cell phenotypes can be broadly subdivided into those that that require low-level antigenic reminders or bona-fide memory cells. For example, CD4+ T cell mediated protection against secondary leishmaniasis requires the persistence of low-level antigenic reminders (48, 49). Persistence of live bacteria in the intestinal draining lymph node is also essential for persistent systemic accumulation of Salmonella-specific CD4+ T cells (50). On the other hand, protective CD8+ T cells with lymphchoriomeningitis virus-specificity persist indefinitely in mice (51, 52). These distinctions beg the question as to whether fetal antigen reminders are necessary for the numerical persistence or functional maintenance of maternal memory T cells with more tolerogenic properties. An interesting consideration in this context may be the increasingly recognized long-term persistence after parturition of fetal microchimeric cells in maternal tissues (53, 54). Are these cells of fetal genetic origin in mothers accidental souvenirs of prior pregnancy or may they be purposefully retained to help reinforce tolerance during next pregnancy with genetically similar offspring? Important next-steps include investigating whether low-level fetal microchimeric cells provide tonic stimulation to sustain tolerance and/or functional exhaustion to memory maternal T cells akin to how maternal microchimeric cells sustain in offspring expanded immunological tolerance to non-inherited maternal antigens (55).

B cells, and their production of immune modulatory cytokines and antibodies, are also dynamically regulated during pregnancy with potentially beneficial or detrimental impacts (56). Progressively increased accumulation of clustered CD27+IgD- switched memory CD19+ B cells and plasmablasts are found at the maternal-fetal interface during human pregnancy (57). These decidual B cells lie in close approximation to FOXP3+ regulatory T cells, selectively produce IL-10, and are phenotypically distinct from circulating B cells – highlighting the potential for cross-talk between these adaptive immune cell types in sustaining fetal tolerance (57, 58). With regards to parity, pregnancy-induced humoral sensitization has long been recognized. A classic example of this is sensitization of Rh(-) women during first pregnancies with Rh(+) offspring that primes accumulation of anti-Rh antibodies causing lysis of Rh(+) fetal red blood cells in subsequent pregnancies (59). The association between the presence and avidity of anti-HLA antibodies during pregnancy, and pregnancy complications including preeclampsia and spontaneous preterm delivery are also highlighted in recent studies (60, 61), but with somewhat discordant impacts on maternal serological responsiveness to fetal antigen stimulation in subsequent pregnancies (62). While these results suggest pregnancy-induced humoral B cell sensitization works in opposition to T cell tolerance, with discordant impacts on fetal-matched tissue organ allografts, being able to stratify the relative roles of B and T cells also opens up the exciting possibility for targeting individual immune components for enforcing fetal tolerance (63).

Emerging data also shows that classical innate immune cells may help mothers remember prior pregnancy and protect against complications in future pregnancies. Natural killer cell subsets that produce IFN-γ and vascular endothelial growth factor accumulate to higher levels in the decidua of multigravid compared with primigravid women (64). Likewise, uterine group 1 innate lymphoid cells expand with accelerated tempo during secondary compared to primary pregnancies in mice and is associated with increased cell-intrinsic expression of the memory chemokine receptor CXCR6 (65). Given the importance of natural killer cells in optimizing placentation that protects against preeclampsia and other pregnancy complications (66–68), “remembering” in the context of parity is likely to include participation by these and other innate immune cell subsets. Important next-steps are to investigate whether partner-specificity controls the relative expansion of these cells at the maternal-fetal interface, and the importance of these innate immune cell subsets for shifts in susceptibility to pregnancy complications between first and subsequent pregnancies.

Together, these results highlighting critical distinctions in the dynamics of immune cells during primary compared with secondary pregnancy in humans and preclinical pregnancy models are likely to be directly relevant in considering how parity and partner-specificity impacts susceptibility to complications in human pregnancy. In turn, many somewhat discordant findings on immunological changes that occur in women during pregnancy or in preclinical animal pregnancy models may be explained by differences in relative importance of specific immune cell subsets and/or the molecules they express during primary compared with subsequent pregnancies. At a minimum, parity and partner-specificity should be important factors in the recruitment and analysis of immunological changes in pregnant women moving forward. In the larger scientific context, understanding the mechanism for how prior pregnancy protects against complications in future pregnancies opens up the exciting possibility that therapeutically mimicking these changes can lead to improved pregnancy outcomes.

## Concluding Remarks

We are currently living in the midst of an unpreceded infectious disease pandemic caused by the novel COVID-19 virus. This unfortunate public health challenge has also unintentionally shown how far modern biomedicine has advanced. Less than 12 months after identification of COVID-19 as a novel coronavirus, multiple formulations of effective vaccines have been developed and are now administered widely across populations. With this infrastructure in place, imagine what can happen if scientists, public health officials, and governments coordinate dedicated resources to tackling the silent pandemic of fetal and infant mortality caused by the great obstetrical syndromes of prematurity, preeclampsia, and stillbirth. The undisputed fact that prior successful term pregnancy protects against a variety of complications in future pregnancy suggests one approach is to simply mimic the physiological changes retained in mothers after pregnancy. Development of vaccines that prime tolerogenic memory instead of effector memory responses to protect against pregnancy complications caused by fetal-intolerance could prove to be a novel approach to combating the pervasive pandemic of stillbirth and infant mortality caused by prematurity that predates, and if not deliberately addressed will certainly outlast, the current COVID-19 virus pandemic.

President John Fitzgerald Kennedy in 1962 challenged us to go to the moon in the current decade, not because it is easy, but because it is hard, emphasizing that “….doing so will serve to organize and measure the best of our energies and skills…. a challenge that we are willing to accept, one we are unwilling to postpone, and one we intend to win.” This mission was accomplished less than eight years later by the amazing crew and extended cast of Apollo 11. There is a lot we still do not understand about how our bodies work. Core knowledge on how our species reproduce and propagate remain rudimentary. With these fundamental gaps in knowledge unaddressed, pregnancy complications will likely remain the leading cause of infant and under age five childhood mortality (69, 70). An estimated 400 babies are born too early (70), another 300 expecting mothers will develop preeclampsia (71), and 75 unborn fetuses in the third pregnancy trimester will have died in utero in the past 15 minutes (72). Is this a challenge we, as a biomedical community, and individual physicians with ambition to generate new knowledge that impacts how we practice medicine, now ready and willing to accept, one we are unwilling to postpone, and one we intend to win? If not, when?

## Data Availability Statement

The original contributions presented in the study are included in the article/supplementary material. Further inquiries can be directed to the corresponding author.

## Ethics Statement

The animal study was reviewed and approved by Cincinnati Children's hospital institutional animal care and use committee.

## Author Contributions

EJG, JL, HM-H, and SSW wrote the paper. JMK performed the experiments. JMK and SSW analyzed the data. All authors contributed to the article and approved the submitted version.

## Funding

This work was supported by the National Institutes of Health, National Institute of Allergy and Infectious Diseases, through grant DP1-AI131080. SSW is supported by the Howard Hughes Medical Institute Faculty Scholars Program, a Burroughs Wellcome Fund Investigator in the Pathogenesis of Infectious Disease Award, and the March of Dimes Ohio Collaborative on Prematurity Research.

## Conflict of Interest

The authors declare that the research was conducted in the absence of any commercial or financial relationships that could be construed as a potential conflict of interest.
